# Structural Insights into Ratite Birds and Crocodile Eggshells for Advanced Biomaterials Design

**DOI:** 10.1021/acsomega.4c10850

**Published:** 2025-01-31

**Authors:** Nerith R. Elejalde-Cadena, Edilberto Hernández-Juárez, Everardo Tapia-Mendoza, Abel Moreno, Lauro Bucio

**Affiliations:** † Laboratorio de Cristalofísica y Materiales Naturales, Instituto de Física, Universidad Nacional Autónoma de México, Circuito de la Investigación Científica S/N, Ciudad Universitaria, Ciudad de México 04510, México; ‡ Laboratorio Nacional de Ciencias para la Investigación y Conservación del Patrimonio Cultural (LANCIC), Instituto de Química, Universidad Nacional Autónoma de México, Ciudad de México 04510, México; § Instituto de Química, Universidad Nacional Autónoma de México, Av. Universidad 3000, Colonia UNAM, Ciudad de México 04510, México

## Abstract

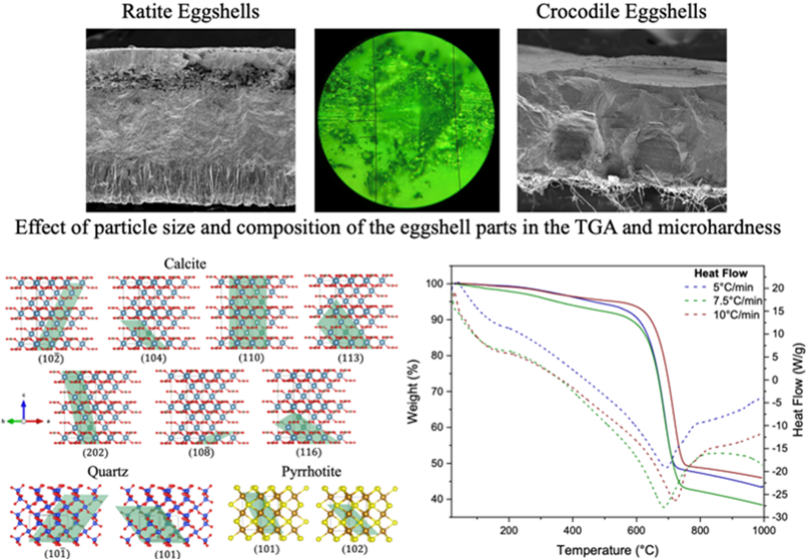

Detailed analysis of particle size, morphology, elemental composition, crystalline structure, and thermal degradation behavior reveals significant differences between ratite and crocodile eggshells, showing their unique environmental adaptations and biological functions. Ratite eggshells, characterized by smaller particle sizes, present lower thermal degradation and are more suitable for applications requiring flexibility and resilience. In contrast, crocodilian eggshells have more extensive and denser particles, giving them a more uniform structure and therefore contributing to their higher thermal stability and mechanical strength. The variation in activation energy profiles between different parts of the eggshells indicates the complexity of their degradation processes. In this regard, ostrich eggshell presents more complicated, multistage thermal degradation patterns, and may be suitable for layered thermal stability applications. In contrast, the uniform degradation behavior of emu eggshells suggests its utility in systems where consistent thermal performance is essential. Similarly, the stable and predictable degradation profiles of river and swamp crocodile eggshells make them ideal candidates for environments requiring high durability and resistance to thermal cycling. This research highlights the natural design of eggshells and provides valuable guidance for the development of biomimetic materials. By mimicking the structural and thermal properties of these eggshells, it would be useful to create thermally stable and mechanical materials suitable for a wide range of industrial and biomedical applications.

## 1 Introduction

Biomaterials are components designed to interact with biological systems for various applications. These materials can be derived from either natural or synthetic sources. They play crucial roles in medicine, industry, agriculture, food technology, materials science, and engineering.^1,2^ The biomineralization process occurs in biological environments, where in an organic matrix, complex biochemical reactions involving organic molecules, such as proteins and polysaccharides, control the nucleation, growth, morphology, and the occurrence of possible polymorphic change within mineral phases that make up the biomineral.^3,4^ These proteins (overexpressed by specific genes) act as nucleators, inhibitors, or surface modifiers that allow the formation of specific crystals. In addition, some organisms could remodel the structure and composition of minerals in their bodies.^5−7^ The ability to synthesize and manipulate minerals is essential for constructing biological structures such as bones, shells, teeth, skeletons, eggshells, and others.^8,9^

Eggshells are solid and porous structures composed primarily of calcium carbonate, magnesium, phosphate, and traces of other elements.^10^ The porosity of eggshells is essential for the life inside the egg, allowing the interchange of matter, providing a suitable homeostatic environment for embryo development.^11^

The process of biomineralization that occurs in the eggshell formation is an interesting case for performing a biomimetic mineralization, which involves imitating nature’s mechanism to develop new materials.^12,13^ Something similar is observed in the processes that use organic hydrogels, which use an amorphous intermediate phase to produce crystalline products that lead to the development of the final mineralized structures.^14−16^

In the formation of eggshells, the process of biomineralization begins with nucleation,^17^ which is the initial phase in which the minerals start to form from an amorphous intermediate phase. In the eggshell, the calcium and carbonate ions bind to form an amorphous calcium carbonate (ACC) precursor.^18^ This step is fundamental because the amorphous state is more flexible and easier to shape within the biological environment, allowing the system to guide where and how the mineralization will occur.^19^ Once nucleation has occurred, the next step is precipitation and stabilization of the amorphous phase by organic molecules or matrices.

The proteins and other organic molecules in eggs control the deposition and growth of calcium carbonate. This phase allows greater control over the final structure, avoiding premature crystallization and ensuring that the minerals are correctly placed.^20,21^ Finally, the deposition of the crystalline products occurs, where the amorphous phase changes to a more stable crystalline form.^22^ In eggshells, this results in the formation of CaCO_3_ calcite, the thermodynamically most stable phase at room temperature, which is essential for the egg’s functioning. This transition is highly regulated by a biological mechanism, which ensures that crystal growth is uniform and correctly aligned to form the shell.^23,24^ Eggshells are solid and porous structures composed primarily of calcium carbonate, magnesium carbonate, calcium phosphate, and minor occurrences of other phases. In biomimetic mineralization, the aim is to imitate this step to produce materials with similar optimized properties, such as strength, durability, and biocompatibility.^25^

The similarity between these processes shows how the natural principles of controlled mineralization can inspire advances in biomaterials, in which biological systems bring innovative solutions in many areas such as materials science, engineering, and medicine.

Although most studies have been focused on hen eggshells, research on ratite (such as ostriches, emus, and rheas) and crocodile eggshells indicates that the different types of eggshells share significant similarities in structure, composition, and morphology. The convergence of information from studies on hen eggshells underscores the importance of exploring and using these natural resources. Despite focusing on hen eggshells, the ratite and crocodile eggshells’ comparable structure, composition, and morphology offer unexploited potential across various fields. By recognizing and exploiting these similarities, advanced materials can be developed that promote sustainability, improve human health, and address contemporary challenges in agriculture, medicine, and industry.

Taking into account all of the considerations previously stated, in this work, we propose to study microhardness, thermogravimetric analysis, particle size, morphology, microstructure, and elemental composition of ratite and crocodile eggshells in order to optimize their applications in various fields to identify unique properties and potential advantages. This would lead to the development of sustainable practices by maximizing the use of natural waste materials. By fully characterizing these eggshells, industries could develop innovative products that reduce waste and dependence on synthetic materials, thus contributing to environmental sustainability.

## 2 Experimental Section

The eggshells of ratites, such as ostrich (*Struthio camelus*) and emu (*Dromaius novaehollandiae*), were obtained from the Sonora market in Mexico City. On the other hand, river crocodile (*Crocodylus acutus*) and marsh crocodile (*Crocodylus moreletti*) eggshells were obtained from the Research Center for the Conservation of Endangered Species (CICEA by its Spanish acronym) of the Juarez Autonomous University of Tabasco. Before use, the eggshells were cleaned with distilled water for 30 min at room temperature to remove any organic residues and contaminants. The membrane was then carefully removed, with care not to break it. Finally, the eggshells were dried at room temperature and used for further measurement. In the case of the crocodile eggshells, the membrane was not adhered to the mineral phase, making it easy to remove it.

### 2.1 Optical Microscopy

Images of the outer and inner surfaces of the eggshells were obtained using an Olympus SZX2-ILLK microscope (Tokyo, Japan) at 4× magnification. DinoCapture 2.0 software was used for image visualization.

### 2.2 Scanning Electron Microscopy and Energy-Dispersive X-ray Spectroscopy (SEM-EDS)

Images were captured with a JEOL JSM-7800F microscope (Peabody, MA) at variable magnification and 15 keV under high vacuum. For elemental composition, an analysis time of 150 s was used. To determine the number of particles, the eggshells’ outer, middle, and inner surfaces were separated. These were pulverized to a powder-like size, and images were taken from each surface. All samples were placed in an aluminum stub, fixed with carbon type, and coated with gold for 30 s to improve conductivity.

The particle counting analysis was conducted at various magnifications to observe the particle size. Due to the irregularity of the particle surfaces and their small size, the data obtained are considered semiquantitative and were cautiously approached. 1000 particles were counted across the four eggshells, with 100 particles examined for each part. Additionally, the particle sizes were measured using ImageJ software.^26^

### 2.3 X-ray Powder Diffraction (XRPD)

Phase identification was performed by using a Bruker D8 Advance diffractometer with Cu-Kα radiation (λ = 1.5406 Å) at a voltage of 35 kV and a current of 25 mA. A Bragg–Brentano configuration was used with a Lynx-eye detector. Data were collected within the 2θ angular range of 4 to 115°, with a step size of 0.019°. Phase identification and crystallographic data were obtained using the American Mineralogist Crystal Structure Database (AMCSD).^27^ The Rietveld refinements were performed using GSAS-II software.^28^

### 2.4 Microhardness

The microhardness of the eggshell surfaces was evaluated by using a Wilson Hardness Tukon 110 microhardness tester. A load of 300 gf was applied for 10 s. For each sample, five measurements were taken from the outer and inner surfaces of each eggshell.^29^ Statistical analysis, such as the *t* test to compare the means of two data series, was performed at a 95% confidence level. In this context, the following statistical hypotheses were formulated: *H*_0_ (null hypothesis): *X̅*_1_ = *X̅*_2_; *H*_1_ (alternative hypothesis): *X̅*_1_ ≠ *X̅*_2_. To accept the null hypothesis (*H*_0_), the value of *t*_experimental_ must be less than the *t*_critical_ value. If *t*_experimental_ is greater than *t*_critical_, the null hypothesis is rejected, and the alternative hypothesis (*H*_1_) is accepted. In the context of this study, accepting the null hypothesis means concluding, with a 95% confidence level, that the means between the analyzed data sets are statistically identical. In contrast, rejecting the null hypothesis indicates, with the same confidence level, that the means are not statistically equal. When performing the *t* test to compare means between two sets of data, the value of the *t*_experimetal_ statistic can vary depending on whether the data are homoscedastic (equal variances) or heteroscedastic (unequal variances). In this study, an *F*-test was performed before the *t* test to determine whether the variances were equal. Consequently, the proposed hypotheses are ; , where *S*_2_^2^ is the variance of the group with the highest variability and *S*_1_^2^ is the variance of the group with the lowest variability. If the null hypothesis is accepted with a 95% confidence level in the *F*-test (*F*_experimental_ < *F*_critical_), this indicates that the variances between the two data sets are equal, which means that the observed variability is homoscedastic. On the contrary, if the alternative hypothesis (*F*_experimental_ > *F*_critical_) is accepted, the variances in the two data sets are not equal and should be considered heteroscedastic data for the *t* test.^30,31^

### 2.5 Thermogravimetric Analysis (TGA)

TG analyses were performed on an SDT Q600 thermal analyzer (TA Instruments) under a nitrogen atmosphere at a flow rate of 50 mL/min flow rate. The temperature scan from room temperature to 1000 °C was developed using alumina crucibles and heating rates of 5, 7.5, and 10 °C/min. The integral isoconversion procedure was performed using the Vyazovkin method to determine activation energies associated with the degradation process without assuming a reaction model.

## 3 Results and Discussion

### 3.1 Characterization of Eggshell Surfaces

**Figure 1:**
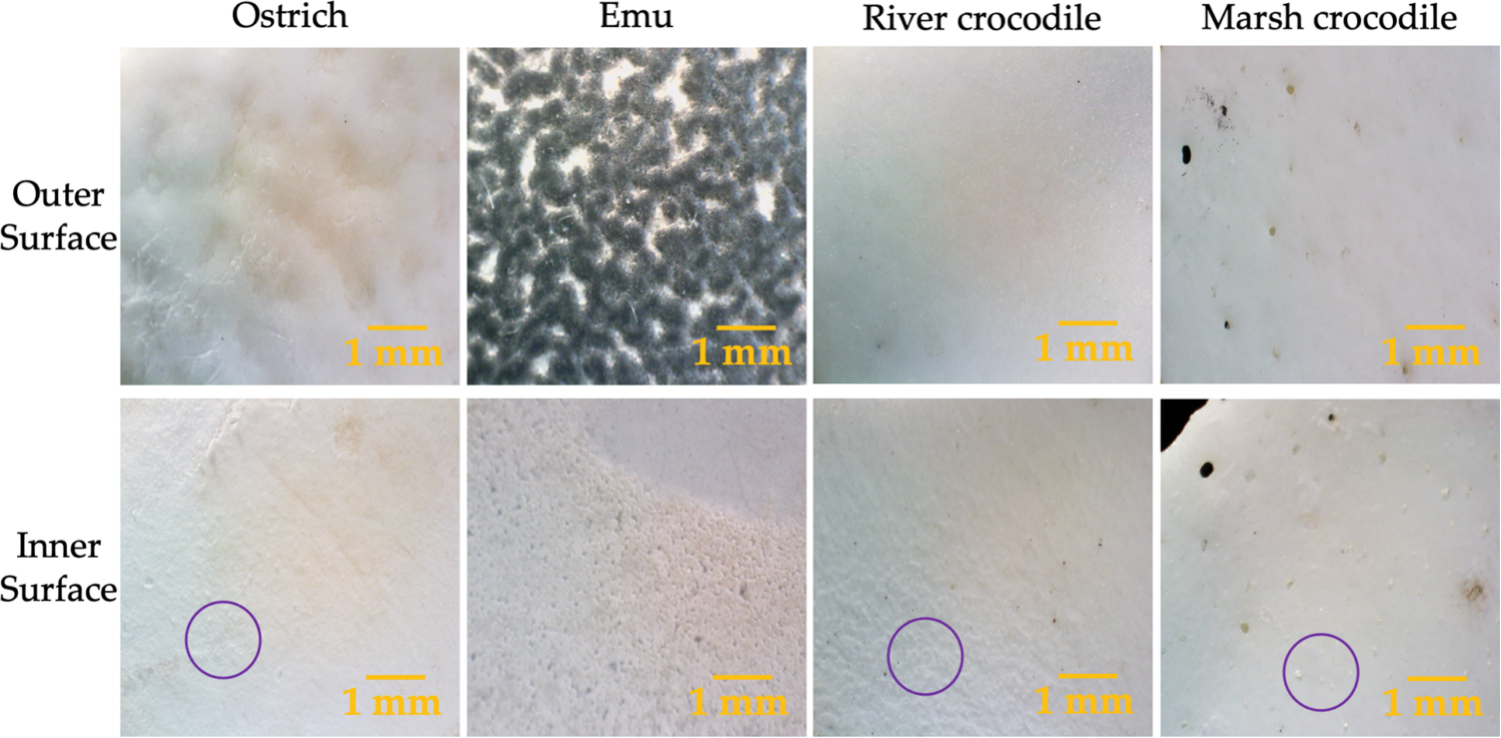
Optical images of the outer and inner surfaces of the eggshells of ratites and crocodiles.

Eggshells vary in morphology, texture, and size depending on the species. Figure 1 shows the outer and inner surfaces of ostrich, emu, river crocodile, and marsh crocodile eggshells. On the outer surface of the ostrich eggshell, a relatively smooth and uniform texture is observed, with slight variations in color, without prominent ornamental patterns. On the other hand, the emu eggshell has a rough texture with a pattern of well-defined dark blue ridges, giving it a more textured appearance compared to the ostrich eggshell. The river and marsh crocodile eggshells present smooth external surfaces. However, the marsh crocodile eggshell shows minor dark spots that could be pores.^32^

The inner surface of the ostrich eggshell has a smooth texture but with some aggregates marked in a purple circle, which could be nucleation sites formed during the eggshell formation process and deposited on the organic membrane.^33,34^ In contrast, the inner surface of the emu eggshell shows a rougher texture, with more visible aggregates distributed over the surface. The inner surface of river and marsh crocodile eggshells is smooth and shows small circular structures marked with a purple circle. These structures are similar to those observed in ratites and could be related to the mineralization process during eggshell formation, a process adapted to the specific needs of each species.

### 3.2 Elemental Composition and Structural Analysis

#### 3.2.1 Elemental Analysis

Scanning electron microscopy and energy-dispersive X-ray spectroscopy (SEM-EDS) analyses were performed on the areas framed by the white line on the outer and inner surfaces of the ratite bird (Figure S1) and crocodile (Figure S2) eggshells. This analysis revealed mainly the presence of calcium, oxygen, and carbon, which correspond to calcium carbonate (mineral) phases. These minerals make the primary structural material of the eggshell, providing the necessary rigidity and strength to protect the developing embryo. The presence of these elements in significant concentrations confirms the well-mineralized nature of the eggshell, which is characteristic of both avian and crocodilian species.^35^ The well-mineralized nature of the eggshells suggests that calcium carbonate is valuable in developing robust and resilient materials.^36,37^

In addition, elements such as sodium, nitrogen, and sulfur were detected in all samples (Table 1). Eggshells contain significant amounts of nitrogen, although present in smaller amounts than calcium and carbon, which play a crucial role in the development and functionality of the eggshell. It is mainly found in intramineral proteins, which actively contribute to biomineralization. These nitrogen-rich proteins regulate the deposition and orientation of calcium carbonate crystals, enhancing the overall structural integrity and strength of the eggshell.^38^ They also play a crucial role in strengthening the eggshell, similar to how bioactive proteins are used in biomaterials to regulate mineralization in bone grafts or tissue engineering.^37^ This knowledge could be applied to develop materials that require precise crystal growth control.^39^ Nitrogen is also associated with the structural and protective function of the eggshell, contributing to the formation of a matrix that supports the deposition of minerals such as calcium carbonate.^40,41^ On the other hand, sulfur, associated with intramineral proteins, is essential to form disulfide bonds within these proteins, which are vital to maintaining the mechanical strength and stability of the eggshell. This is comparable to how cross-linking agents in synthetic polymers enhance the durability of biomaterials.^42,43^ The role of sulfur in structure and function facilitates biomineralization and provides the mechanical integration necessary for embryo protection.^33^

**Table 1:** EDS Analysis: Elemental Composition of Ratite Birds and Crocodile Eggshells^a^

	ostrich		emu		river crocodile		marsh crocodile	
element	outer	inner	outer	inner	outer	inner	outer	inner
O	40.02 ± 0.66	26.80 ± 0.87	28.75 ± 0.92	39.74 ± 0.91	34.06 ± 1.33	32.34 ± 1.46	36.22 ± 1.33	36.41 ± 1.31
C	24.60 ± 0.57	48.49 ± 1.20	40.24 ± 1.14	17.54 ± 0.58	28.02 ± 1.18	37.22 1.58	23.55 ± 1.06	31.55 ± 1.24
Ca	33.41 ± 0.48	8.03 ± 0.29	15.06 ± 0.46	37.24 ± 0.78	19.51 ± 0.74	13.03 ± 0.63	23.15 ± 0.81	13.29 ± 0.55
Mg	0.43 ± 0.09	--	--	--	--	--	--	--
Na	1.17 ± 0.13	3.42 ± 0.18	3.61 ± 0.19	1.28 ± 0.13	8.09 ± 0.42	6.97 ± 0.42	6.81 ± 0.37	9.75 ± 0.45
K	--	--	0.44 ± 0.10	--	--	--	--	--
N	--	10.63 ± 1.84	10.35 ± 1.87	4.20 ± 1.56	10.32 ± 2.29	9.19 ± 2.82	10.27 ± 2.20	9.00 ± 2.23
S	--	2.24 ± 0.15	0.32 ± 0.10	--	--	1.25 ± 0.20	--	--
Cl	--	0.39 ± 0.09	--	--	--	--	--	--
Al	--	--	0.30 ± 0.08	--	--	--	--	--
Si	0.37 ± 0.08	--	0.93 ± 0.09	--	--	--	--	--

a The data is given in %.

Another element identified in both ratite and crocodilian eggshells is sodium. In birds, this element influences the structural and functional integrity of the eggshell by controlling the ionic balance and interactions between organic and inorganic components.^44^ This feature is similar to its use in biomaterials like hydrogels, which help to maintain the performance in biological environments.^43,45^ In crocodiles, sodium reflects structural needs, environmental factors, and maternal dietary influences, indicating an adaptive response in eggshell composition to external conditions.^40^

In addition to these primary elements, magnesium, potassium, chlorine, aluminum, and silica were identified in deficient concentrations (Table 1). Despite their low concentrations, these elements could play specific roles in the structure, stability, and function of the eggshell and they could be used in biomaterials to adjust mechanical properties to improve structural support.^46^

The presence of potassium and magnesium, mainly on the external surface, would contribute to the modulation of mechanical properties and the stability of the crystalline structure.^47^ Aluminum and silica are also found on the external surfaces, enhancing the eggshell’s structural framework. Chlorine, detected only on the inner surface of ostrich eggshell, is believed to influence ionic bonds with other minerals, affecting the overall mineralization process.^48^

#### 3.2.2 Morphology of the Structures

**Figure 2:**
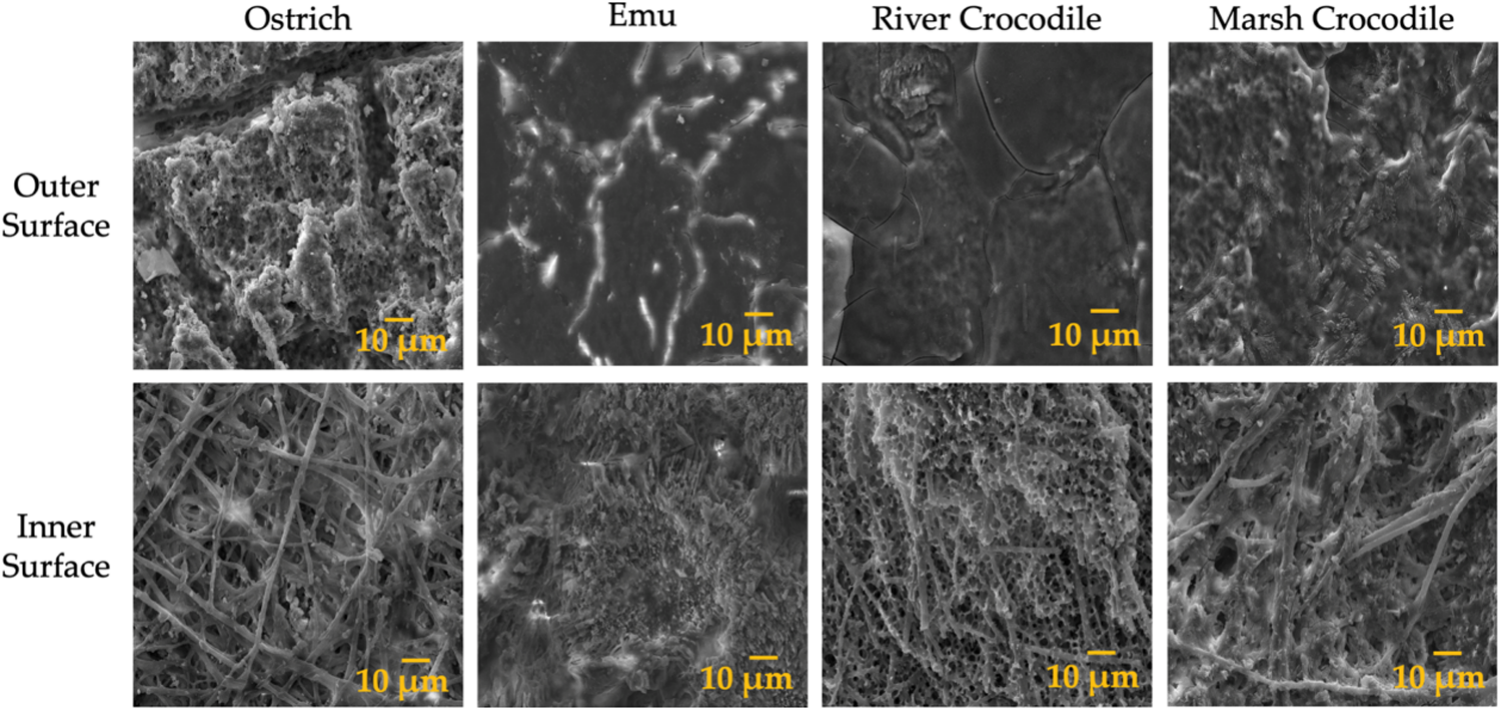
SEM images of the outer and inner surfaces of the ratite and crocodile eggshells.

SEM images of the external and internal surfaces of ratite and crocodile eggshells (Figure 2) provide a detailed comparison of structural features. The outer surface of the ostrich eggshell has an irregular and porous texture with a lattice-like pattern. This porosity is essential for gas exchange and other physiological processes. It also has ridges that contribute significantly to the mechanical strength and structural integrity of the eggshell. On the other hand, emu eggshells have a smoother outer surface with fewer visible pores than ostrich eggshells, indicating less porosity and a more uniform structure. This morphology is adapted to birds’ lifestyles and provides an optimal balance between strength and lightness for incubation and embryo protection.^35^

In contrast, the river and marsh crocodile eggshells have a relatively smooth outer surface with minimal porosity, indicating a more compact structure and resulting in greater strength and protection against external factors. This adaptation is consistent with the nesting environment of crocodiles, where they face different challenges than birds, such as the need to protect eggs in an aquatic environment.^49^

In the case of the inner surfaces, the ostrich eggshell has a more fibrous surface due to an organic matrix that favors the deposition of calcium carbonate and provides flexibility to the eggshell. In the case of the emu eggshell, a denser network of particles is observed, probably given a higher degree of mineralization, which contributes significantly to the mechanical strength and rigidity of the eggshell. Something different is observed on the inner surface of the crocodile eggshells, where a higher density of fibers is obtained, more compact and with fewer visible pores, further contributing to the overall mechanical resistance of the mineral phase.^44,50,51^

In addition, this surface is usually softer than the outer surface, which could facilitate the egg’s movement along the different sections of the fallopian tube without causing damage to the developing embryo. This softness and fibrous structure helped to protect the embryo during development and maintain the structural integrity of the egg during incubation.^52,53^

Differences in eggshell structure between species reflect diverse adaptations to environmental and biological needs, offering insights into the design of advanced biomaterials.

**Figure 3:**
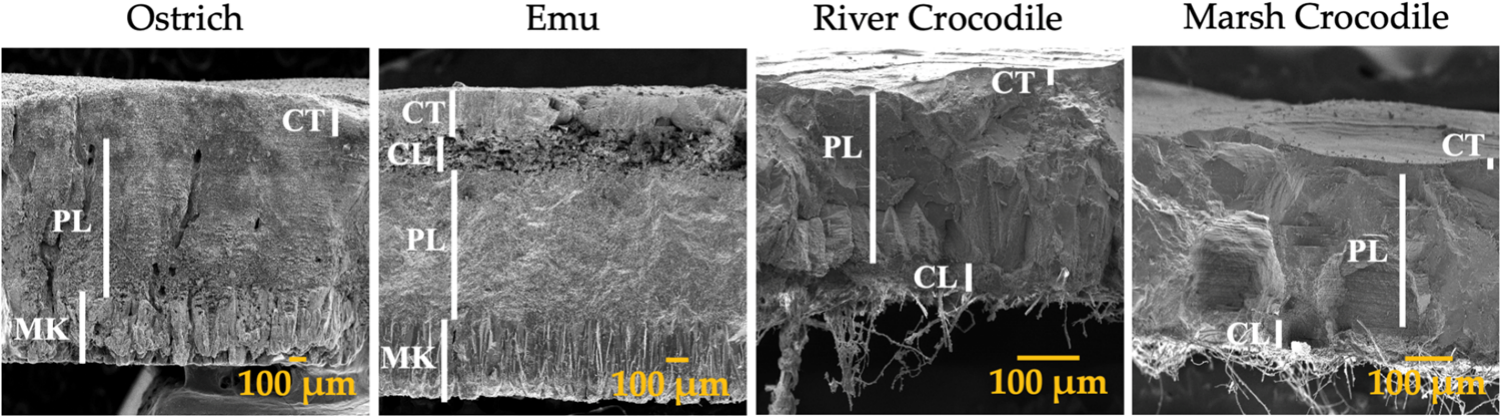
SEM images of the lateral parts of ratite birds and crocodile eggshells. M: Membrane; MK: Mammillary knobs; PL: Palisade layer; CL: Vertical crystal layer; CT: Cuticle.

Finally, in the cross section (Figure 3), structural differences are evident between the eggshells of ratites and crocodiles. Ratite eggshells show a divided and well-organized structure where each part is discernible. These layers include the mammillary knobs (MK), which serve as foundational points for calcification; the palisade layer (PL), known for its dense and compact arrangement of crystals; the vertical crystalline layer (CL), which provides additional structural support; and the cuticle (CT), which offers protection and facilitates the gas exchange.^44^ In contrast, crocodile eggshells have a different structural composition, characterized by the absence of mammillary knobs. The cuticle of crocodile eggshells is remarkably thin, which may be an adaptation of their specific reproductive environment. Below the cuticle, the eggshell consists mainly of a palisade layer and a crystalline layer, contributing to the rigidity and durability of the eggshell. The lack of mammillary knobs in crocodile eggshells simplifies the overall structure but still provides adequate protection and support for the developing embryo.^50,51^

A notable difference is observed between ostrich and emu eggshells in the ratite group. The palisade layer in the ostrich eggshell is slightly thicker than that in the emu, suggesting a difference in mechanical properties, possibly related to differences in the environmental conditions or incubation strategies. In addition, the emu eggshell has an additional crystalline layer between the cuticle and the palisade layer, a feature not present in the ostrich eggshell. This crystalline layer in the emu may contribute to increased mechanical strength and play a role in specific adaptations of the species.

#### 3.2.3 Particle Counting

**Figure 4:**
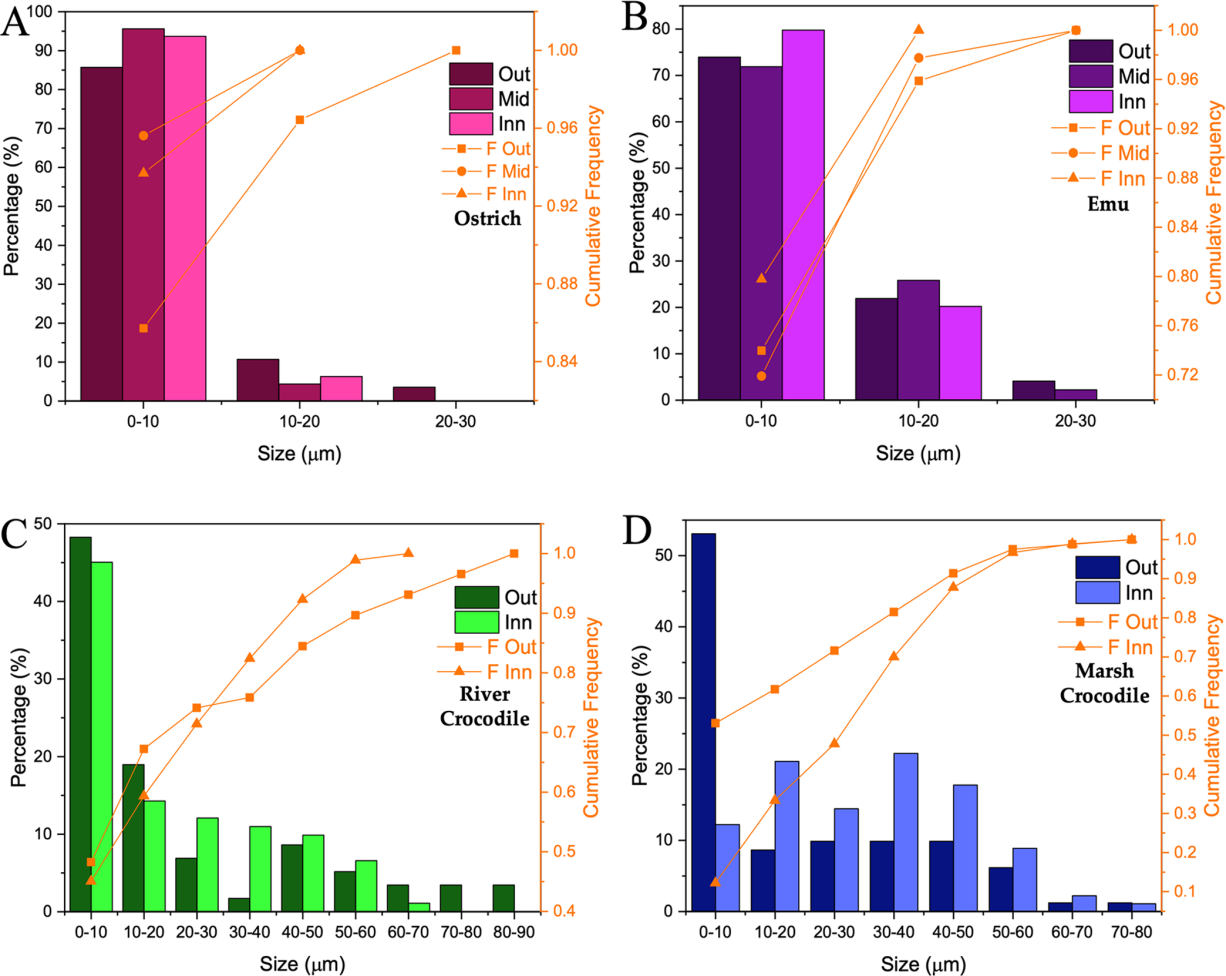
Particle size distribution of ostrich (A), emu (B), river crocodile (C), and marsh crocodile (D) eggshells. Out: Outer part; Mid: Middle part; Inn: Inner part; F: Cumulative frequency.

Particle size analysis of ratite and crocodile eggshells revealed a broad range from approximately 1 to 90 μm (Figure 4). Specifically, the particle size distribution in ratite eggshells was more limited, ranging from approximately 1 to 30 μm (Table S1). These particles exhibited an anhedral structure with poorly defined morphologies that tended to form prismatic shapes (Figure S3). In the ostrich eggshell, the middle and inner parts contain particles smaller than those of the outer layer (Figure 4A). In contrast, in the emu eggshell, the middle part was characterized by larger particles relative to the inner part (Figure 4B).

Significant variations in particle size were observed within crocodilian eggshells, ranging from approximately 1 to 90 μm (Table S2). Unlike the more homogeneous size distribution found in the ratite eggshells, the distribution in crocodile eggshells was less uniform. These particles exhibited a subhedral structure with morphologies resembling prismatic forms (Figure S4). In the eggshell of the river crocodile, the inner part contained particles smaller than those in the outer part (Figure 4C). In contrast, the eggshell of the marsh crocodile exhibited particles of similar size in both the inner and outer parts (Figure 4D).

Furthermore, a cumulative frequency of particle sizes revealed that particles within crocodile eggshells have similar dimensions in both the outer and the inner parts. This uniformity in particle size distribution suggests more significant variability and a lower incidence of voids between particles, contributing to a much more compact structure. In contrast, ratite eggshells show a narrower particle size distribution, facilitating the formation of more voids, which influences the porosity of the material and the formation and distribution of pores. Consequently, ratite eggshells probably possess higher porosity than crocodile eggshells, developing thinner pores and a higher pore frequency.

The observed differences in particle size distribution and morphology between ratite and crocodile eggshells are directly related to how these materials could inspire the design of biomaterials with controlled porosity and mechanical properties. Ratite eggshells, with their narrower particle size distribution and possible higher porosity, suggest potential applications in biomaterials where high porosity is advantageous, such as in scaffolds for tissue engineering.^54−56^

Conversely, the varied particle size distribution in crocodile eggshells and their more uniform structure and possibly lower porosity suggest that they could inspire the design of biomaterials that will require greater mechanical strength and durability. The compact structure with fewer voids in crocodile eggshells can be emulated in biomaterials intended for load-bearing applications or protective coatings, where minimizing porosity and maximizing structural integrity is essential.^46,57,58^

### 3.3 Crystalline Phase Identification

**Figure 5:**
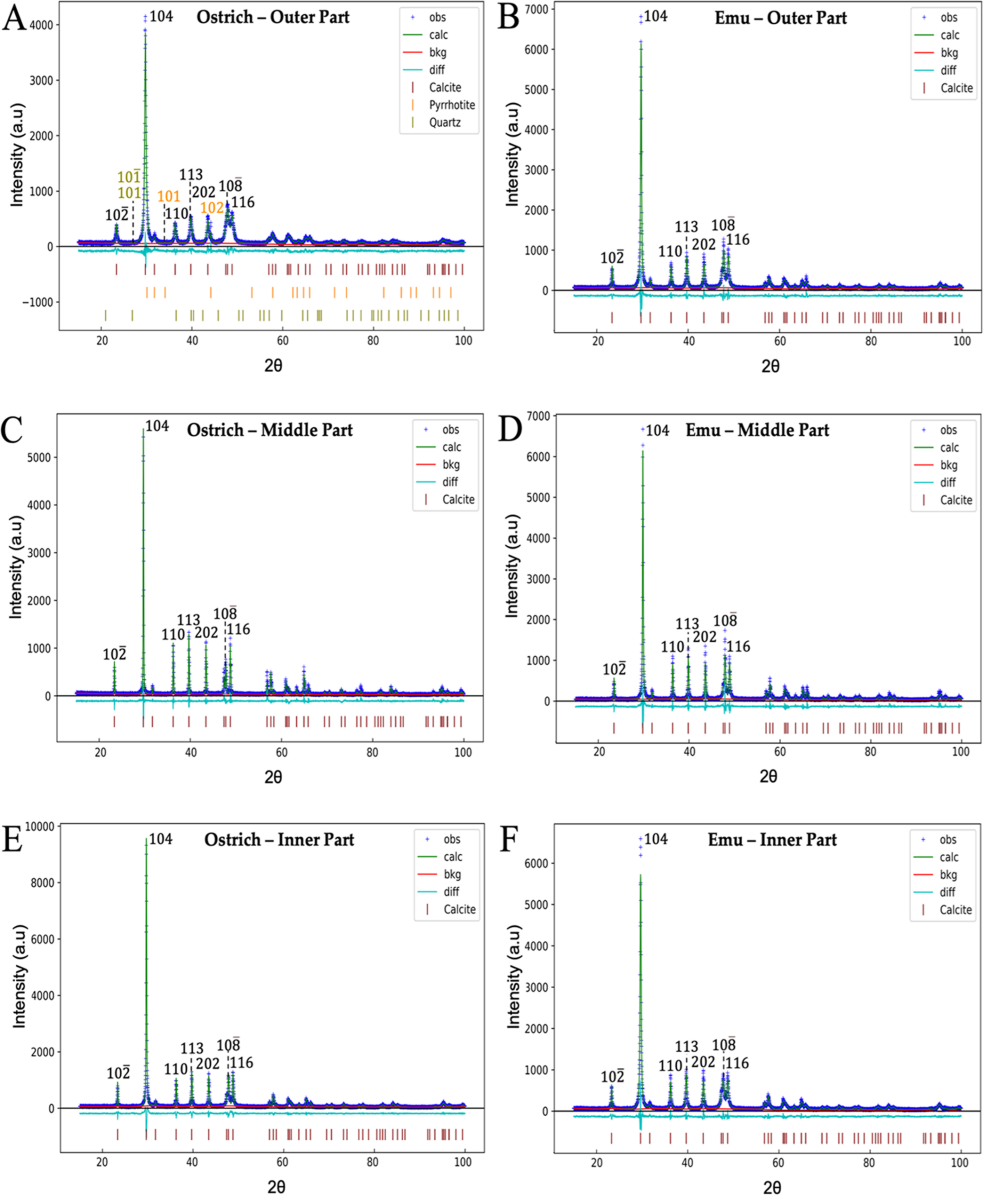
Diffraction patterns of the outer, middle, and inner parts of ostrich (A, C, E) and emu (B, D, F) eggshells. The light brown and orange numbers correspond to the quartz and pyrrhotite *d_hkl_* planes.

**Figure 6:**
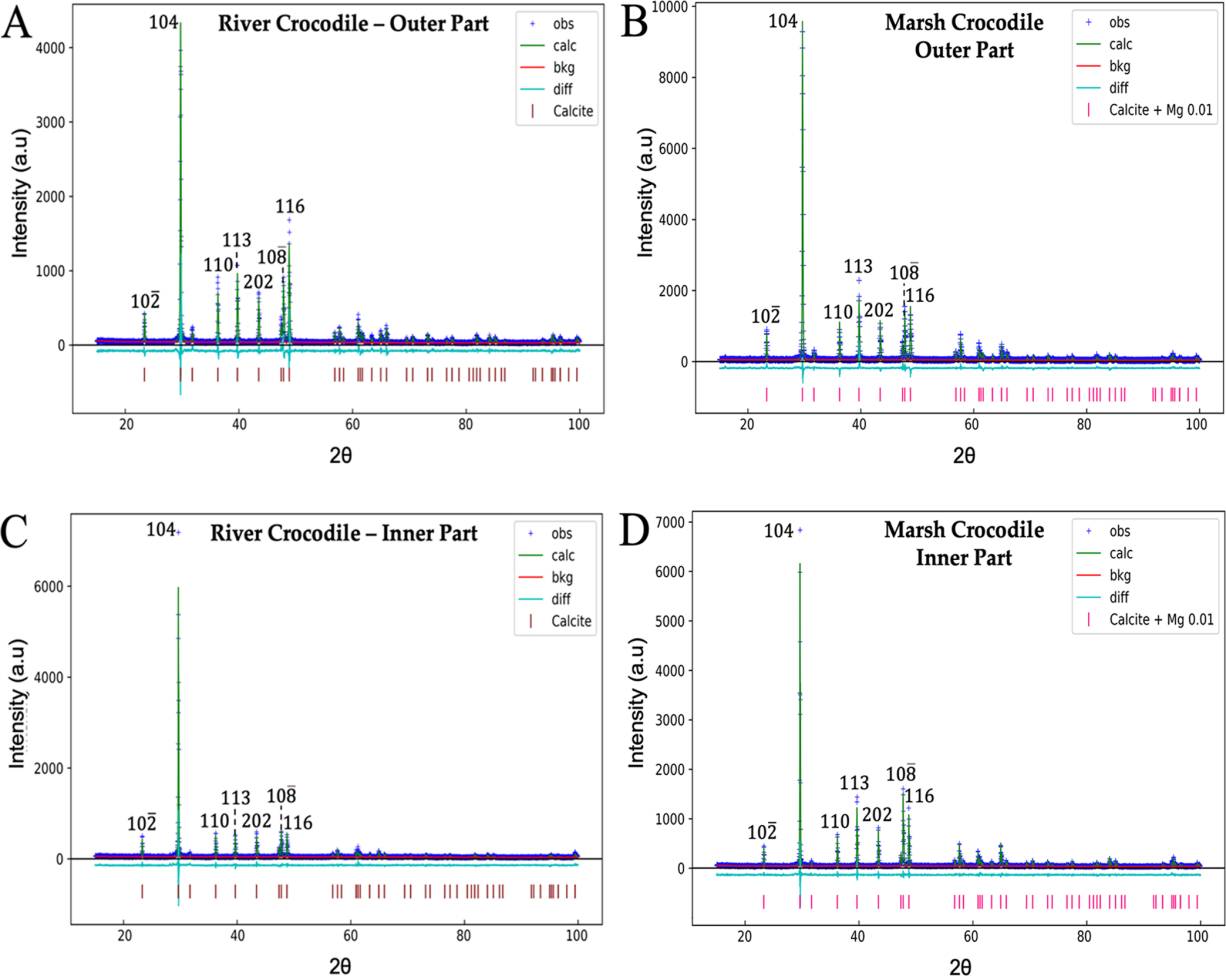
Diffraction patterns of the outer and inner parts of river (A, C) and marsh (B, D) crocodile eggshells.

Rietveld refinements obtained from XRD have provided information on the crystalline phases’ composition in the diffractograms of ratite and crocodile eggshells (Figures 5 and 6). In the emu eggshell, the only crystalline phase identified was CaCO_3_ calcite. On the other hand, in the ostrich eggshell, in addition to calcite, two other crystalline phases are identified on the outer surface, corresponding to SiO_2_ quartz and Fe_0.95_S_1.05_ pyrrhotite (Table S3). The diffraction planes of calcite are assigned to a trigonal space group *R*3̅*c* with cell parameters *a* = 4.986 Å and *c* = 17.049 Å.^59^ The (101) and (101̅) diffraction planes associated with the quartz phase showed a preferred orientation that allowed the identification of this phase as well as the planes (101) and (102) for pyrrhotite (Figure 5).^60,61^

In the crocodile eggshells (Figure 6), only the crystalline phase corresponding to calcite was identified. However, in the marsh crocodile eggshell, calcite has a magnesium content of 1% (Ca_0.99_Mg_0.01_CO_3_) (Table S4), which could affect the mechanical strength and other physical properties of the eggshell.^62^

Identifying quartz and pyrrhotite in ostrich eggshells suggests a variability in surface mineralization not observed in emu and crocodile eggshells. Several factors, including environmental conditions, dietary intake, or evolutionary adaptations, may influence this mineralogical distinction. This suggests that ostrich eggshell have gone through different mineralization processes, which could lead to unique functional properties that are not present in the eggshells of other species.^36,63^

Furthermore, magnesium-modified calcite in the marsh crocodile eggshell emphasizes the significant impact that minor element inclusions could have on the crystal structure of the eggshells. This modification is probably the result of biological adaptations or specific environmental conditions during the formation of the eggshells. Incorporating magnesium into the calcite could alter the crystalline properties of the eggshells, improving their mechanical strength, durability, and resistance to environmental stressors.^64,65^

### 3.4 Microhardness Characterization

Vickers microhardness measures the surface resistance of a material on a microscopic scale. This technique is used to measure the hardness of materials, especially those that are too small or thin for conventional hardness testing. Vickers microhardness uses a much smaller load instead of a large load, as in traditional hardness testing, making it ideal for small, thin, or fragile samples. It is also helpful for characterizing heterogeneous or composite materials, as it can measure hardness at specific locations within a sample. In addition, this technique is less sensitive to the crystalline orientation of the material than other hardness tests, making it more versatile for characterizing materials with complex crystal structures.^66,67^

Statistical estimates, including mean, standard deviation, and variance, are presented in Table S5. When the microhardness measurements for ratite birds are compared, differences in mean values are observed. This indicates a difference in microhardness between the inner and outer surfaces of ratite eggshells. However, this disparity is less evident when the inner and outer surfaces of crocodilian eggshells are compared in terms of their mean values.

Statistical results were obtained after four comparisons of the microhardness means. The outer surfaces were compared with the inner surfaces of ratite birds and crocodile eggshells (Table 2). The results indicate that the analysis between the surfaces of ostrich and marsh crocodile eggshells presented homoscedastic data, while emu and river crocodile eggshells presented heteroscedastic data. However, when the *t*-values calculated for microhardness were analyzed, it was found that there were significant differences between the outer and inner surfaces of each eggshell. This suggests that in terms of microhardness the outer surfaces of the eggshells have a different microhardness than the inner surface. It is important to note that for the ratites and the river crocodile, the inner surfaces had a higher microhardness than the outer surfaces. On the other hand, in the marsh crocodile eggshell, the outer surface had a higher microhardness. Therefore, it is suggested that this change may be influenced by the presence of magnesium in the calcite phase, a feature not observed in the eggshell of the river crocodile.

**Table 2:** Statistical Values of *F* and *t*-Test Takin Data of Microhardness for the Outer and Inner Surfaces of Ratite Birds and Crocodile Eggshells

	ratite bird				crocodile			
	ostrich		emu		river		marsh	
	outer	inner	outer	inner	outer	inner	outer	inner
1	78.42	165.63	55.42	76.76	67.16	103.61	45.10	135.32
2	73.85	156.67	58.50	90.76	74.77	115.86	58.37	107.15
3	77.69	149.67	57.61	84.03	58.86	137.85	45.38	105.70
4	81.89	153.80	58.38	110.79	47.74	140.71	87.07	127.33
5	85.73	169.70	55.60	94.70	53.38	166.32	77.90	102.74
variance	20.222	69.480	2.233	163.979	116.030	587.664	363.352	215.313
*F*_exp_	3.436		73.444		5.065		0.593	
*F*_crit_	6.388		6.388		3.482		6.388	
result	*F*_exp_ < *F*_crit_		*F*_exp_ > *F*_crit_		*F*_exp_ > *F*_crit_		*F*_exp_ < *F*_crit_	
(test-*F*)	*H*_0_		*H*_1_		*H*_1_		*H*_0_	
*t*_exp_	18.788		5.950		6.110		–4.916	
*t*_crit_	±2.447		±2.776		±2.447		±2.306	
result	*t*_exp_ > *t*_crit_		*t*_exp_ > *t*_crit_		*t*_exp_ > *t*_crit_		*t*_exp_ > *t*_crit_	
(test-*t*)	*H*_1_		*H*_1_		*H*_1_		*H*_1_	

In addition, four other comparisons were made. First, the outer surface of the ostrich eggshell was compared with the outer surface of the emu eggshell. In a second comparison, the difference between the inner surfaces of the ostrich eggshell and emu eggshell was analyzed. Finally, two more comparisons, identical to those described above, were made using river crocodile and marsh crocodile eggshells (Table 3). From this analysis, it was found that the relationship of the outer surfaces of the ratites presented heteroscedastic data.

**Table 3:** *F*-Test and *t*-Test Statistics Microhardness between the Outer and Inner Surfaces of Eggshells of Ostrich, Emu, River Crocodile (C), and Marsh Crocodile

	outer surface				inner surface			
	ostrich	emu	river C	swamp C	ostrich	emu	river C	marsh C
1	78.42	55.42	67.16	45.10	165.63	76.76	103.61	135.32
2	73.85	58.50	74.77	58.37	156.67	90.76	115.86	107.15
3	77.69	57.61	58.86	45.38	149.67	84.03	137.85	105.70
4	81.89	58.38	47.74	87.07	153.80	110.79	140.71	127.33
5	85.73	55.60	53.38	77.90	169.70	94.70	166.32	102.74
variance	20.222	2.233	116.030	363.352	69.480	163.979	587.664	215.313
*F*_exp_	9.057		2.360		3.131		2.729	
*F*_crit_	6.388		6.388		3.633		6.256	
result	*F*_exp_ > *F*_crit_		*F*_exp_ < *F*_crit_		*F*_exp_ < *F*_crit_		*F*_exp_ < *F*_crit_	
(test-*F*)	*H*_1_		*H*_0_		*H*_0_		*H*_0_	
*t*_exp_	10.577		–9.901		0.243		1.359	
*t*_crit_	±2.571		±2.306		±2.306		±2.306	
result	*t*_exp_ > *t*_crit_		*t*_exp_ > *t*_crit_		*t*_exp_ < *t*_crit_		*t*_exp_ < *t*_crit_	
(test-*t*)	*H*_1_		*H*_1_		*H*_0_		*H*_0_	

Regarding the *t* test, it was determined that there is no difference in the means of the measurements of the inner surfaces with 95% confidence. This indicates that the microhardness of the inner surfaces is similar between species. However, for the outer surfaces, the microhardness is statistically different, indicating that the microhardness varies between the species. Specifically, the outer surface of the ostrich eggshell had a higher microhardness, as did the outer surface of the marsh crocodile.

### 3.5 Calcium Carbonate Decarbonation

**Figure 7:**
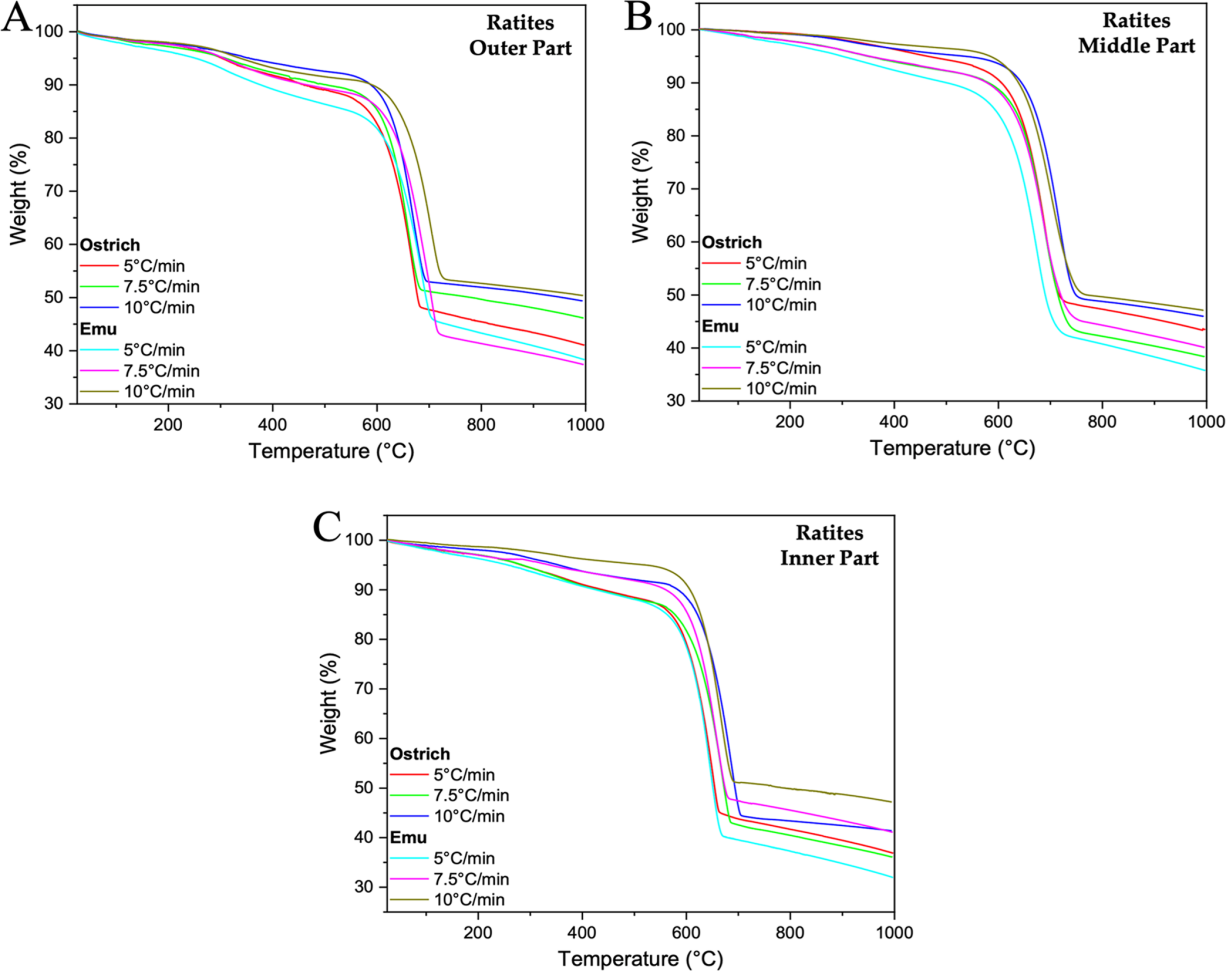
TG graphs of the outer (A), middle (B), and inner (C) parts of ratite birds’ eggshells.

**Figure 8:**
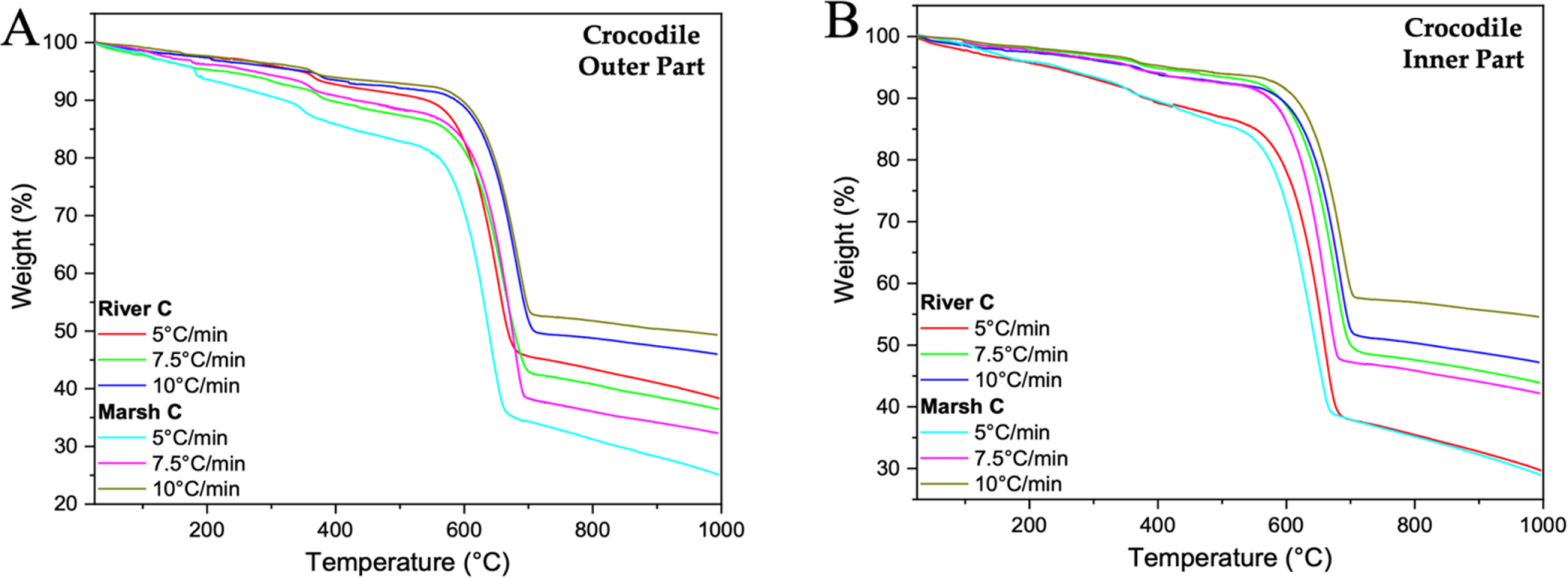
TG graphs of the outer (A) and inner (B) parts of the crocodile eggshells.

Thermogravimetric (TG) curves illustrating the thermal degradation process of calcite within the eggshells of ratites and crocodiles are shown in Figures 7 and 8. Analysis of these curves provides information about both surface and structural water loss during heating. The initial weight loss observed between approximately 50 and 130 °C corresponds to the loss of surface-bound water molecules. This is followed by a more significant reduction between 130 and 300 °C, corresponding to the release of water structurally integrated within the calcite matrix.^68^ The influence of water loss is particularly pronounced on the outer and inner surfaces of the eggshells of ratites and crocodiles, with notable effects at heating rates of 5 and 7.5 °C/min. This highlights the sensitivity of the eggshell to varying thermal conditions, particularly water content, which plays a critical role in the integrity and stability of the calcite structure during the degradation process.^69^

The weight percentages of the surface and structural water contents in the eggshells vary depending on the species. For ratite eggshells, ostrich eggshell contains surface and structural water ranging from 5 to 13%, while emu eggshell contains between 4 and 15%. In comparison, the eggshell of river crocodile and marsh crocodile contains 8–14% and 6–18%, respectively (Table S6). These variations indicate that water is a critical factor in preserving the structural integrity of eggshells under different environmental conditions. Understanding how the water content influences the stability and function of these natural materials is essential for developing biomaterials designed to perform reliably under various thermal conditions. Furthermore, the different hydration profiles observed between species reflect their environmental adaptations and underline the importance of water in preserving eggshell properties. This knowledge could be critical in designing biomaterials that are thermostable and resilient in diverse environments.^70,71^

The decomposition of calcium carbonate occurs within the temperature range of about 550 to 700 °C, depending on the heating rates applied. The decomposition percentages of CaCO_3_, as described in Table S6, vary between 36 and 51%. This aligns closely with those reported in previous studies by Petkova et al.^72^

For pure CaCO_3_, an ideal weight loss of 44% is expected due to the decarbonation process. Furthermore, an intriguing trend is observed with increasing heating rates: the maximum temperature (*T*_m_) (Table S7) in the differential thermal gravimetry curves (DTG) progressively changes to higher values (Figures S5 and S6). This change can be attributed to decreased heat transfer efficiency within the system, as noted by Radojevic et al.^73^

A detailed understanding of thermal properties, including water loss, thermal degradation, and calcium carbonate decomposition, provides a basis for using ratite and crocodile eggshells in biomaterials. For example, the ability of these materials to maintain structural integrity under varying thermal conditions could be exploited in developing biomaterials that require thermal stability, such as protective coatings or components in heat-resistant applications.^74,75^ The differences in water content and thermal behavior between ratite and crocodile eggshells could inform the design of biomaterials with specific hydration profiles, optimizing them for use in environments where moisture content and temperature fluctuations are important.^76,77^

Besides, the observed trends in thermal degradation and the influence of minor elements such as magnesium on the calcite structure suggest the possibility of adapting biomaterials to improve mechanical strength and durability. By mimicking the natural processes observed in these eggshells, it may be possible to create advanced biomaterials that offer high thermal stability and robust mechanical properties suitable for various industrial and biomedical applications.^78,79^

**Figure 9:**
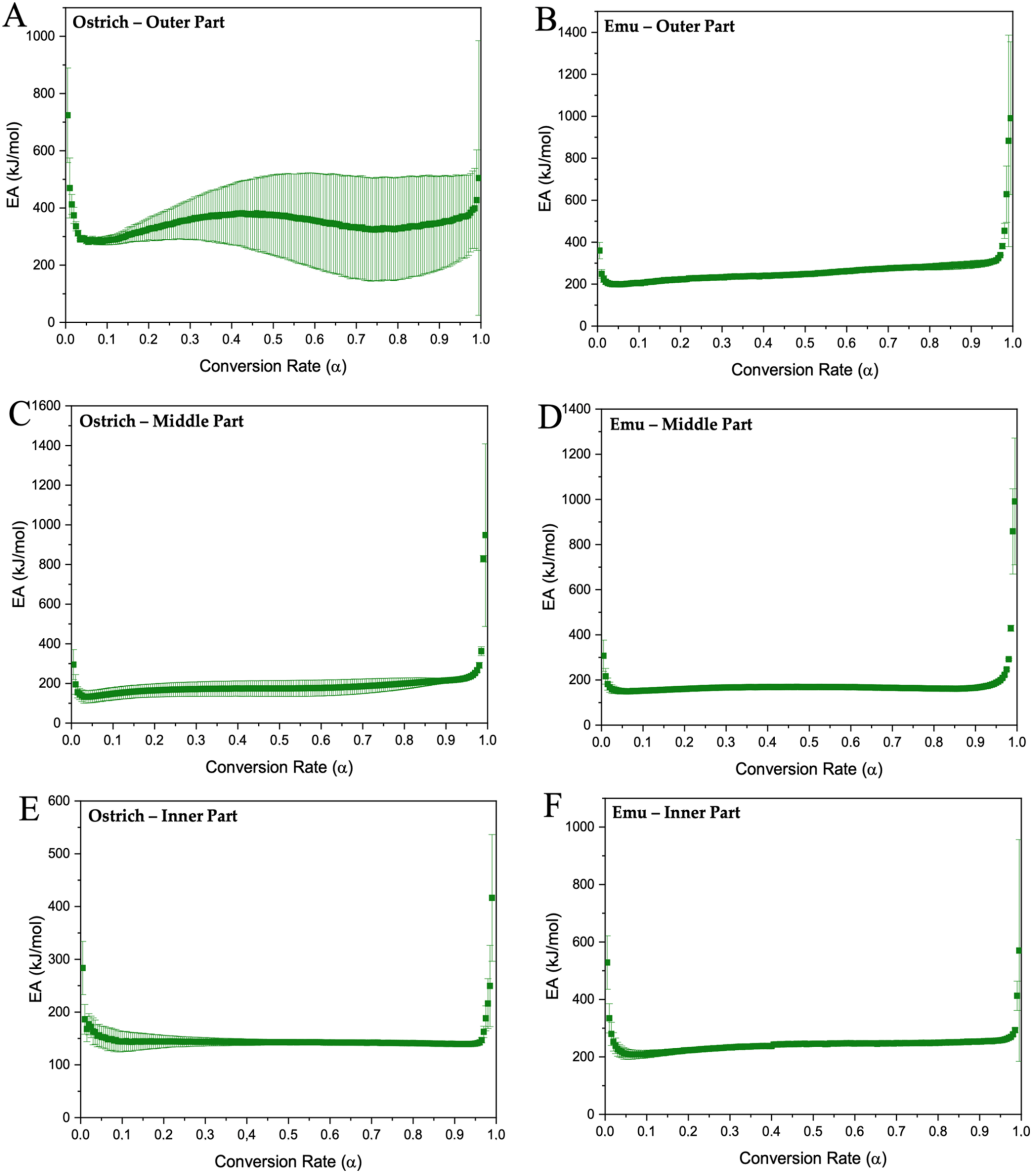
Activation energy graphs of outer, middle, and inner parts of eggshells of ostrich (A–E) and emu (B, D, F).

**Figure 10:**
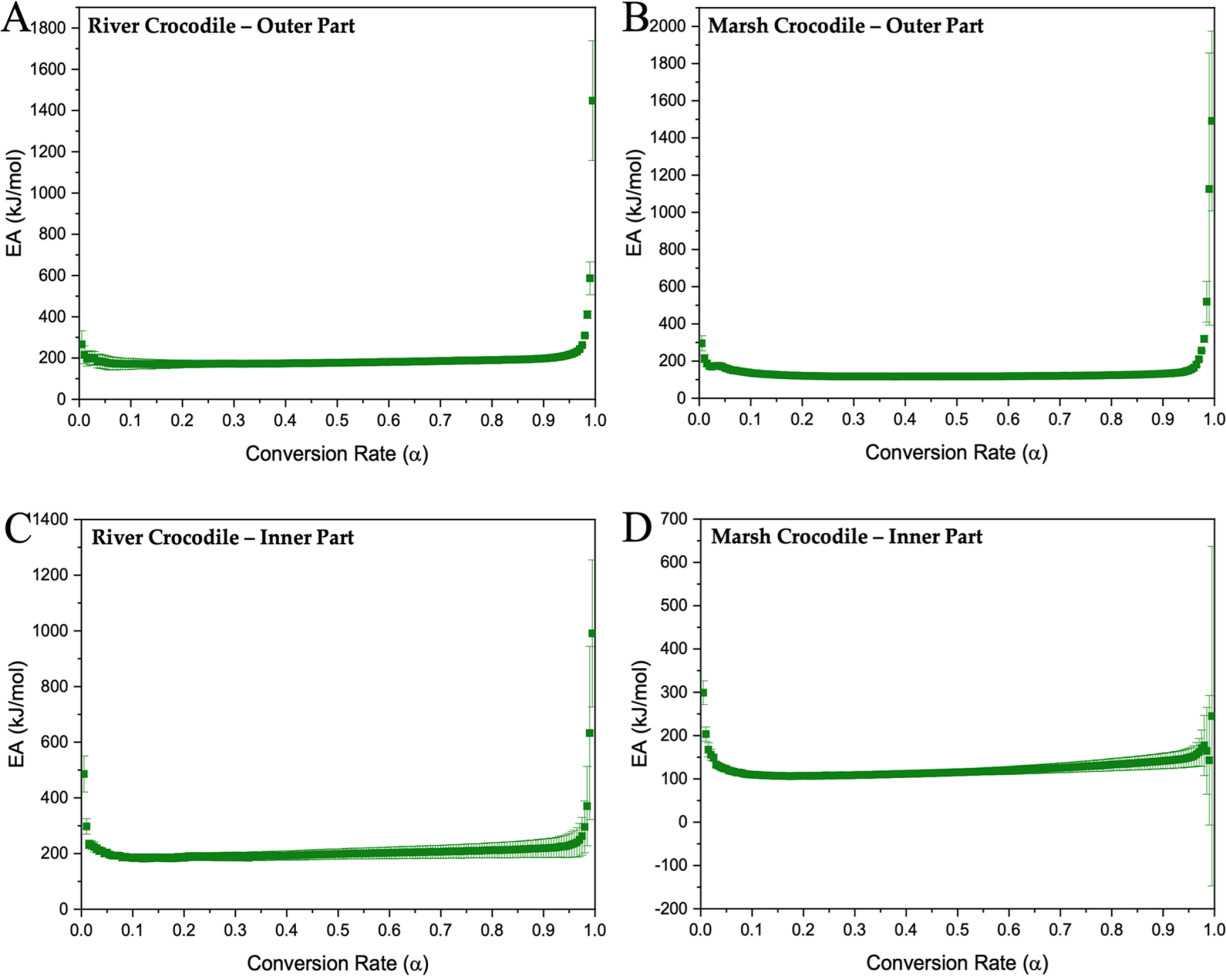
Activation energy graphs of outer and inner parts of eggshells of river (A, C) and marsh (B, D) crocodiles.

In addition, Vyazovkin’s approach^80^ establishes an algebraic relationship that effectively models the interaction between the conversion rate and activation energy at each process stage. It also recognizes the impact of the physical properties of the medium on the reaction, considering them part of the energy barrier.^81^ The initial phase of calcium carbonate degradation is particularly complex, reflecting the multistep nature of the process, as evidenced by the TG curves (Figures 7 and 8). This complexity significantly influences the activation energy. In all analyzed samples, the conversion rate shows a variation (Figures 9 and 10), which indicates that the kinetics process involved in the carbonate degradation process within eggshells is multistep. The complexity is especially pronounced at the beginning of degradation, where diffusion-controlled dehydration reactions lead to the initial weight loss observed in the TG curves (Figures 7 and 8). These reactions lead to fluctuations in activation energy, linked to the dehydration and subsequent degradation of hydroxyl groups of Ca(OH)_2_, followed by the decomposition of CaCO_3_.^82^ As the process continues, the calcite, depending on its degree of mineralization, gradually releases CO_2_, resulting in the formation of CaO as a byproduct through the reduction of carbon at elevated temperatures.

Following Vyazovkin’s nonlinear model,^80^ activation energy data were obtained for the eggshell parts from ratites and crocodiles, as illustrated in Figures 9 and 10. The activation energy for the outer part of the ostrich eggshell (Figure 9A) initially decreases to 312 kJ/mol and then stabilizes, followed by a slight increase as the conversion rate approaches one until reaching an energy of 374 kJ/mol. This pattern indicates a complex degradation process, probably involving several stages in which different components decompose at different rates. The gradual stabilization followed by an increase is probably due to quartz and pyrrhotite, which would require more energy to degrade in later stages, especially pyrrhotite.^69,83,84^

The middle and inner parts of the ostrich eggshell (Figure 9C,9E) exhibit a more stable activation energy during most of the conversion process (133 and 163 kJ/mol, respectively), with a significant increase only near complete conversion (252 kJ/mol) for the case of the middle part. However, in the inner part, the energy remains constant (164 kJ/mol), which indicates a uniform degradation process, probably due to a more homogeneous composition. The increase at the end suggests the presence of highly stable components that only degrade at high temperatures.^85^

Like the ostrich eggshells, all parts of emu eggshells show low and stable activation energy over most of the conversion range (202, 198, and 227 kJ/mol for the outer, middle, and inner parts), with a significant increase only near the end (339, 223, and 267 kJ/mol, respectively) (Figure 9). This uniformity suggests that the eggshell has a homogeneous composition in all parts. A similar case is observed in crocodile eggshells, with stable activation energy during most of the conversion process (198 and 217 kJ/mol for the outer and inner parts of the river crocodile and 173 and 133 kJ/mol for the marsh crocodile) and a gradual increase toward the end (243 and 210 kJ/mol for the outer parts of the river and marsh crocodile and 249 and 162 kJ/mol for the inner parts) (Figure 10).

Also, the variation of activation energy concerning the conversion rate highlights the complex nature and multiple steps of the calcium carbonate pyrolysis process. This variation may be related to various simultaneous reactions occurring within the sample and the formation of gaseous products. These results suggest that the decomposition process follows a multistep kinetic mechanism in which thermal degradation is not a single-step reaction but rather a series of reactions that collectively contribute to the overall process.^86,87^

The activation energy profiles of ostrich and emu eggshells reveal significant differences in their thermal stability and degradation behavior, which are crucial for their possible applications as biomaterials. With their different activation energy profiles and increases in later stages of degradation, ostrich eggshells appear more suited for applications requiring materials that can resist higher temperatures or offer gradual thermal resistance.^35,69^ In contrast, the more uniform activation energy profiles of emu eggshells, particularly in the middle and inner parts, indicate their suitability for applications needing homogeneous thermal behavior and predictable degradation rates. The low and stable activation energy across the conversion process suggests that emu eggshells could be ideal for controlled-release systems in biomedical applications, where a uniform degradation process is necessary.^88,89^ The strong increases in the activation energy near the end of the conversion process in ostrich and emu eggshells suggest that the final stages of degradation involve more stable components, which could be exploited in the design of biomaterials to create compounds that maintain structural integrity until the final stages of degradation, ensuring controlled performance over prolonged periods.^90,42,58^

Similarly, the activation energy profiles of river and marsh crocodile eggshells highlight their potential as stable and reliable materials in biomaterial applications, particularly in environments requiring thermal resistance and mechanical durability.^29,71^ The low and stable activation energies during most of the conversion process in both the outer and middle parts of these crocodile eggshells could indicate a consistent composition and predictable degradation behavior. This uniformity benefits biomaterial applications where controlled and reliable performance is important, such as in slow-degrading implants or protective coatings that must maintain integrity over time. The increase in activation energy at the end of the degradation process, as observed in ratite eggshells, also suggests that the crocodile eggshells contain stable components that resist decomposition until higher temperatures are reached, which makes them particularly suitable for high-temperature environments or components that must resist thermal cycling, ideal for load-bearing applications or situations requiring long-term durability.^40,74^

## 4 Conclusions

Analysis of ratite and crocodile eggshells reveals a complex interplay among particle size, morphology, elemental composition, crystal structure, thermal degradation, and microhardness, all of which contribute to the unique properties of these eggshells. Ratite eggshells, characterized by smaller, more uniformly distributed particles and higher porosity, exhibit lower thermal stability, as higher porosity facilitates the loss of surface water at lower temperatures, leading to more early thermal degradation, as observed in TG analyses. This porosity could be correlated with lower microhardness since the material’s structure is less compact and less resistant to mechanical stress. This is probably due to its anhedral, less compact structure and the absence of reinforcing elements, such as magnesium. These characteristics make ratite eggshells more flexible and resilient in specific environments but less durable under thermal and mechanical stress.

In contrast, crocodile eggshells show more varied particles with a denser subhedral crystal structure enhanced by the presence of magnesium in the calcite matrix. This results in high thermal stability and microhardness, allowing these eggshells to maintain their structural integrity under more extreme conditions. The well-ordered crystalline arrangement and reduced porosity of crocodile eggshells contribute to their mechanical strength, providing excellent resistance to mechanical deformation and delaying thermal degradation by slowing heat penetration.

The unique properties of these eggshells not only highlight the evolutionary adaptations of these species but also serve as inspiration for biomaterial development. The intricate interplay of structural and compositional factors observed in crocodile and ratite eggshells could be the basis for the design of advanced materials for applications requiring tailored mechanical strength, thermal stability, and flexibility. By emulating these natural systems, the creation of innovative materials with potential applications in fields such as biomedicine, engineering, and aerospace, among others, could be carried out, taking advantage of nature’s engineering principles.

## Supporting Information Available

SEM-EDS images, as well as tables of particle size percentages, crystallographic data for each of the phases, statistical data used in the microhardness analysis, information related to decarbonation percentages, and TG-DTG graphs for each of the eggshells (PDF)
