# Supplementary material for: Structural Insights into Ratite Birds and Crocodile Eggshells for Advanced Biomaterials Design
Source: ACS Omega. 2025 Jan 31;10(5):5064–79. doi: 10.1021/acsomega.4c10850 (PMC11822711; doi:10.1021/acsomega.4c10850)
Supplement: Supplementary file 1 — ao4c10850_si_001.pdf [file ao4c10850_si_001.pdf]

Structural Insights into Ratite Birds and Crocodile Eggshells for Advanced Biomaterials Design

Nerith R. Elejalde-Cadena,♠ Edilberto Hernández-Juaréz,♠ Everardo Tapia-Mendoza,♦ Abel Moreno,♣ Lauro Bucio♠**

♠ Laboratorio de Cristalofísica y Materiales Naturales, Instituto de Física, Universidad Nacional Autónoma de México, Circuito de la Investigación Científica s/n, Ciudad Universitaria, Ciudad de México, 04510, México.

♦ Laboratorio Nacional de Ciencias para la Investigación y Conservación del Patrimonio Cultural (LANCIC), Instituto de Química, Universidad Nacional Autónoma de México, Ciudad de México, México.

♣ Instituto de Química, Universidad Nacional Autónoma de México, Av. Universidad 3000, Ciudad de México, 04510, México.

* Corresponding authors: rocioec@fisica.unam.mx - bucio@fisica.unam.mx

SUPPORTING INFORMATION

S1. Elemental composition of eggshells of ratites and crocodiles

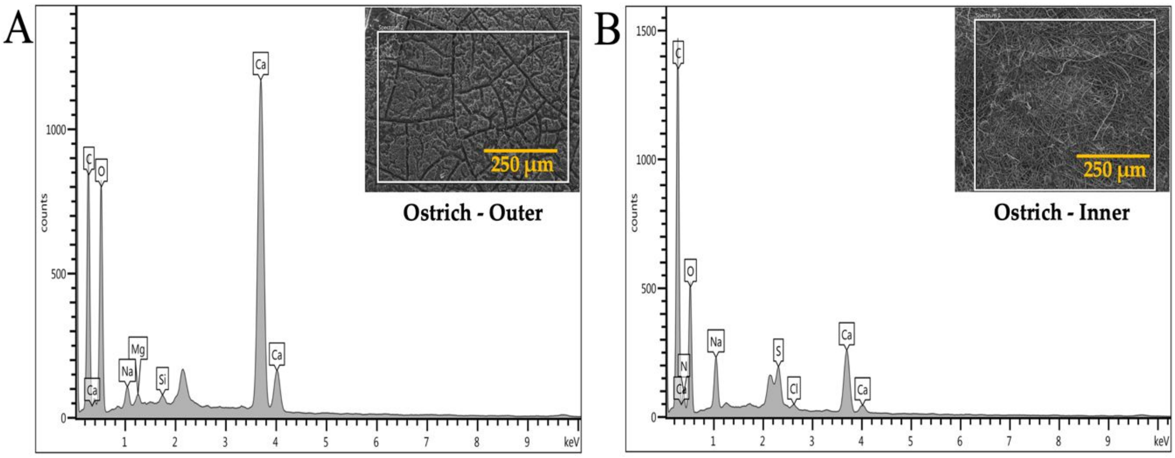

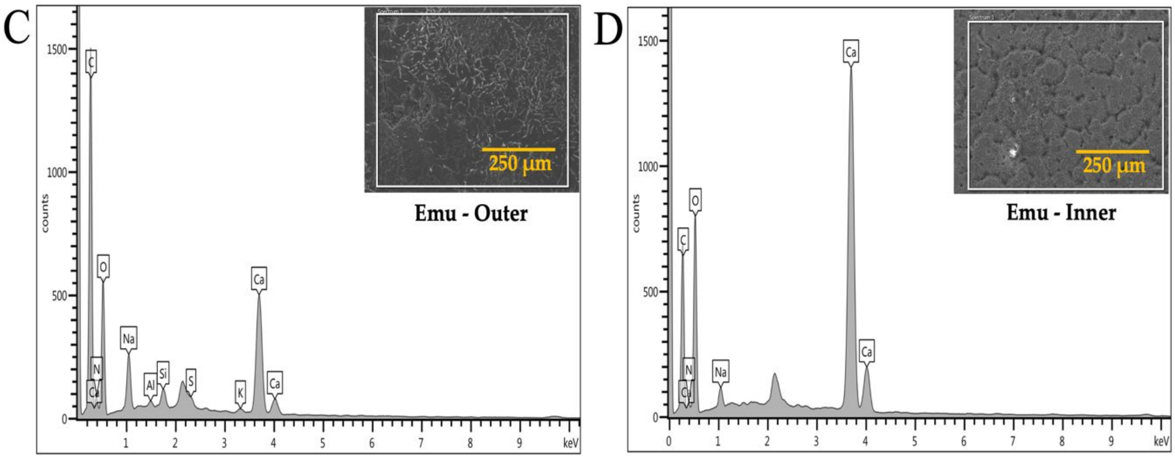

Figure S1. SEM-EDS images of the area outlined in white correspond to the analysis performed on the outer and inner surfaces of ostrich (A, B) and emu (C, D) eggshells.

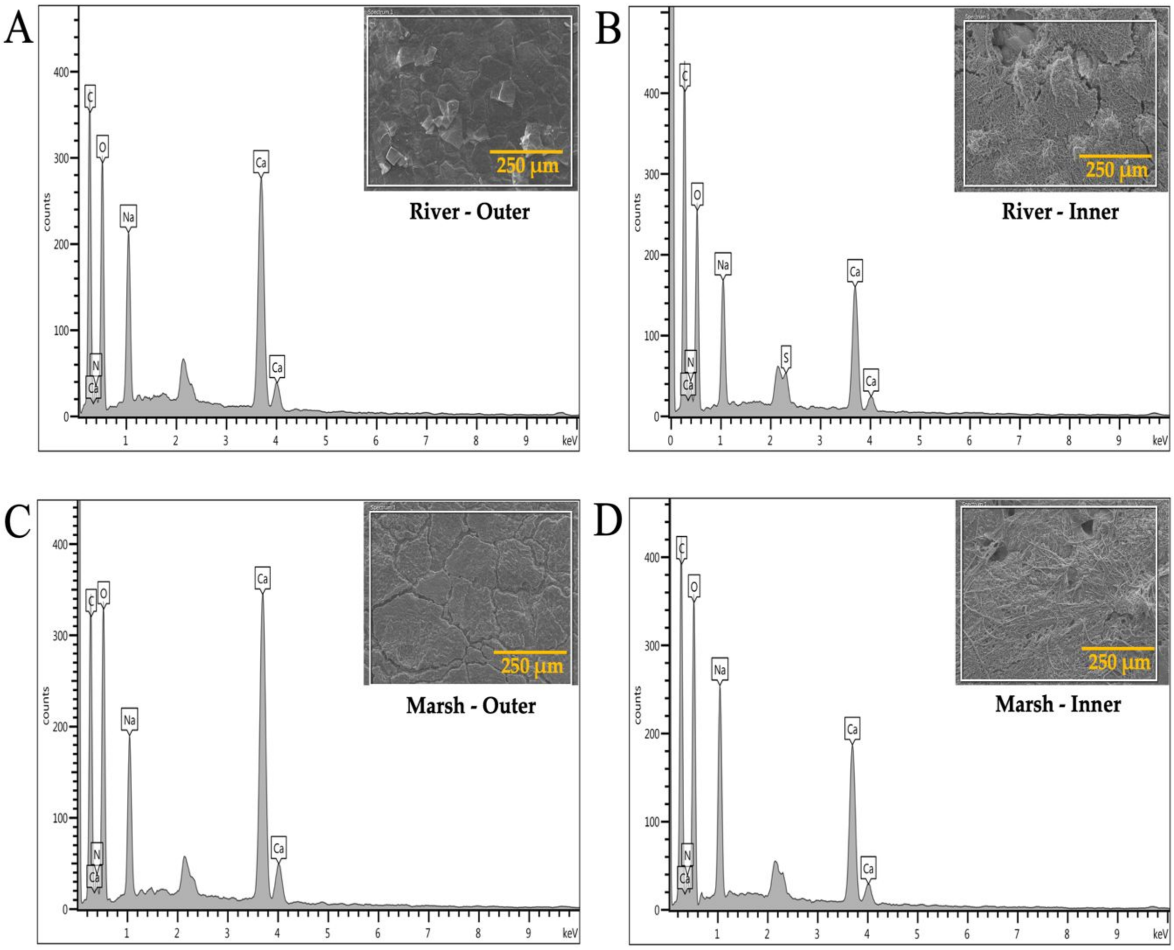

Figure S2. SEM-EDS images of the area outlined in white correspond to the analysis performed on the outer and inner surfaces of river crocodile (A, B) and marsh crocodile (C, D) eggshells.

S2. Particle size distribution obtained by SEM

Table S1. Particle size distribution of ratite eggshells. Out: Outer part; Mid: Middle part; Inn: Inner part; CF: Cumulative frequency*

Size (µm)	Ostrich						Emu					
	Out*	CF Out	Mid*	CF Mid	Inn*	CF Inn	Out*	CF Out	Mid*	CF Mid	Inn*	CF Inn
0 – 10	85.71	0.86	95.61	0.96	93.68	0.94	73.97	0.74	71.91	0.72	79.79	0.80
10 – 20	10.71	0.96	4.39	1	6.32	1	21.92	0.96	25.84	0.98	20.21	1
20 – 30	3.57	1	--	--	--	--	4.11	1	2.25	1	--	--

*The data are given in %

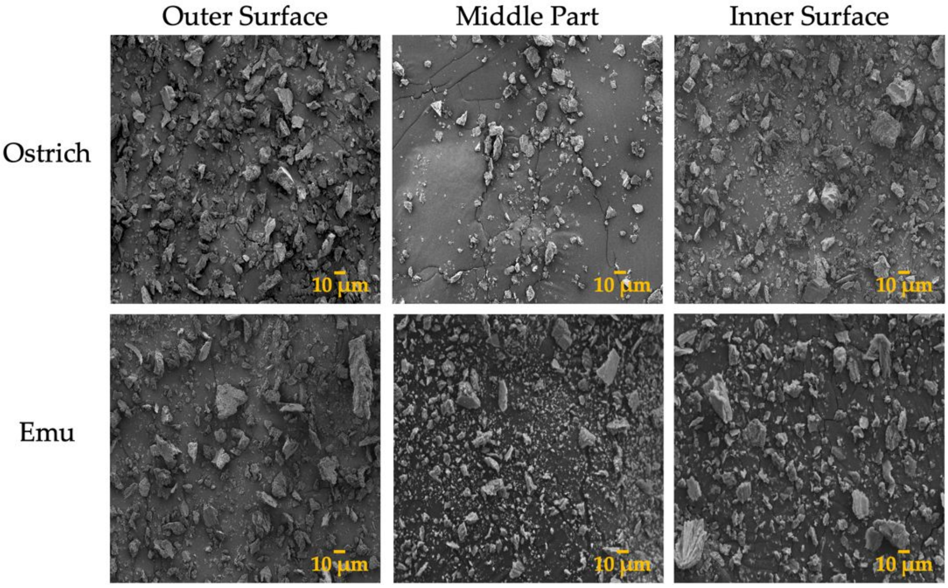

Figure S3. SEM images of the size and particle distribution of ratite eggshells.

Table S2. Particle size distribution of crocodile eggshells. Out: Outer part; Mid: Middle part; Inn: Inner part; CF: Cumulative frequency*

Size (µm)	River crocodile				Swamp crocodile			
	Out*	CF Out	Inn*	CF Inn	Out*	CF Out	Inn*	CF Inn
0 – 10	48.28	0.48	45.05	0.45	53.09	0.53	12.22	0.12
10 – 20	18.97	0.67	14.29	0.59	8.64	0.62	21.11	0.33
20 – 30	6.90	0.74	12.09	0.71	9.88	0.72	14.44	0.48
30 – 40	1.72	0.76	10.99	0.82	9.88	0.81	22.22	0.70
40 – 50	8.62	0.84	9.89	0.92	9.88	0.91	17.78	0.88
50 – 60	5.17	0.90	6.59	0.99	6.17	0.98	8.89	0.97
60 – 70	3.45	0.93	1.10	1	1.23	0.99	2.22	0.99
70 – 80	3.45	0.97	--	--	1.23	1	1.11	1
80 – 90	3.45	1	--	--	--	--	--	--

*The data are given in %

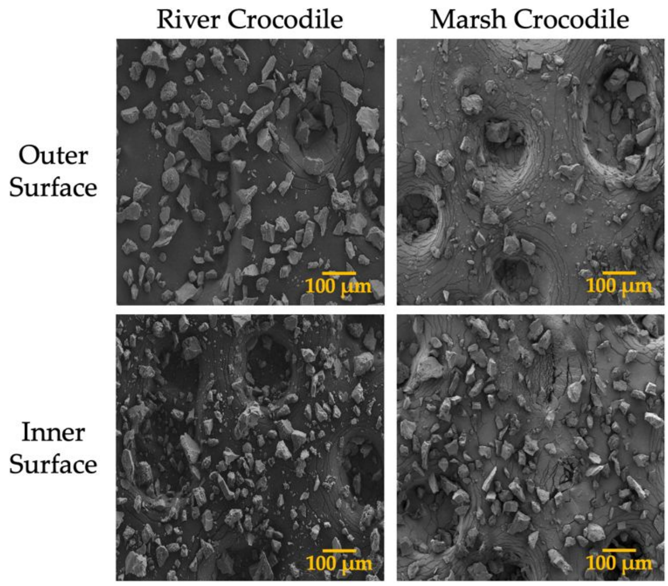

Figure S4. SEM images of the size and particle distribution of crocodile eggshells.

S3. Crystallographic analysis of the phases of ratite birds and crocodile eggshells

Table S3. Summary of the Rietveld refinement for the crystalline phases present in eggshells of ratite birds.

Sample	Ratite Birds		
Identified phases	Calcite	Quartz	Pyrrhotite
Crystal system	Trigonal	Trigona	Hexagonal

							1	
Space group	R-3c						$P3_2 21$	P 6_3/mmc
Z	6						3	4
**Refinement results**	**Emu**			**Ostrich**			**Ostrich**	
**Sample label**	**Outer**	**Middle**	**Inner**	**Outer**	**Middle**	**Inner**	**Outer**	**Outer**
a (Å)	4.9880	4.9883	4.9850	4.9791	4.9878	4.9852	4.9484	3.4537
c (Å)	17.0693	17.0645	17.0495	17.0303	17.0597	17.0483	5.3681	5.5796
V (Å^3)	367.797	367.746	366.929	365.645	367.557	366.927	113.843	58.679
Dcalc (g/cm^3)	2.711	2.712	2.718	2.727	2.713	2.718	3.858	4.909
Harmonic order*	6	6	10	10	8	10	0	6
wR (%)	12.257	16.241	12.402	11.929	15.474	12.834	11.929	11.929
GoF	1.36	1.64	1.35	1.29	1.35	1.43	1.29	1.29

*Preferred orientation model: Spherical harmonics

Table S4. Summary of the Rietveld refinement for the crystalline phases present in eggshells of crocodiles.

**Sample**	**Crocodiles**			
Identified phases	Calcite		Calcite + 0.1 Mg	
Crystal system	Trigonal		Trigonal	
Space group	R-3c		R-3c	
Z	6		6	
**Refinement results**	**River Crocodile**		**Marsh Crocodile**	
**Sample label**	**Outer**	**Inner**	**Outer**	**Inner**
a (Å)	4.9859	4.9836	4.9856	4.9855
c (Å)	17.0380	17.035	17.0442	17.0444
V (Å^3)	366.818	366.423	366.896	366.888
D_{calc} (g/cm^3)	2.719	2.721	2.675	2.675
Harmonic order*	12	14	4	12
wR (%)	18.803	15.679	15.231	14.302
GoF	1.56	1.22	1.45	1.18

*Preferred orientation model: Spherical harmonics

S4. Analysis of microhardness of eggshell surfaces of ratites and crocodilians

Table S5. Statistical data of eggshells of ratite birds and crocodiles*

**Ratite Birds**		**Crocodile**	
**Ostrich**	**Emu**	**River**	**Marsh**

	Outer	Inner	Outer	Inner	Outer	Inner	Outer	Inner
**1**	78.42	165.63	55.42	76.76	67.16	103.61	45.10	135.32
**2**	73.85	156.67	58.50	90.76	74.77	115.86	58.37	107.15
**3**	77.69	149.67	57.61	84.03	58.86	137.85	45.38	105.70
**4**	81.89	153.80	58.38	110.79	47.74	140.71	87.07	127.33
**5**	85.73	169.70	55.60	94.70	53.38	166.32	77.90	102.74
**Mean**	79.516	159.094	57.102	91.408	60.382	132.870	62.764	115.648
**Stn. Dev**	4.497	8.336	1.494	12.805	10.772	24.242	19.062	14.674
**Variance**	20.222	69.480	2.233	163.979	116.030	587.664	363.352	215.313

*The data are given in Hv

S5. Decarbonation of calcium carbonate present in eggshells of ratites and crocodiles

Table S6. Percentage mass loss of the eggshell parts from ratite birds and crocodiles at different heating rates. O: Outer; M: Middle; I: Inner*

	**Ratite Birds**						**Crododiles**			
	**H_2O**									
	**Ostrich**			**Emu**			**River**		**Swamp**	
**T (^oC)**	**O**	**M**	**I**	**O**	**M**	**I**	**O**	**I**	**O**	**I**
5	11.56	7.68	11.94	14.60	10.42	12.47	10.14	13.76	17.43	14.24
7.5	10.33	9.61	12.16	11.59	8.58	9.06	14.00	7.55	11.98	7.71
10	7.85	5.84	8.65	9.29	4.09	5.62	8.95	8.38	7.70	6.44
	**$CaCO_3$**									
	**Ostrich**			**Emu**			**River**		**Swamp**	
**T (^oC)**	**O**	**M**	**I**	**O**	**M**	**I**	**O**	**I**	**O**	**I**
5	40.49	43.84	43.40	39.94	47.46	47.48	43.92	48.41	48.20	47.14
7.5	38.38	47.36	44.98	45.61	45.47	43.46	43.42	43.82	50.52	45.06
10	39.22	44.93	47.02	37.38	45.95	43.31	41.40	40.34	39.78	36.02

*The data are given in %

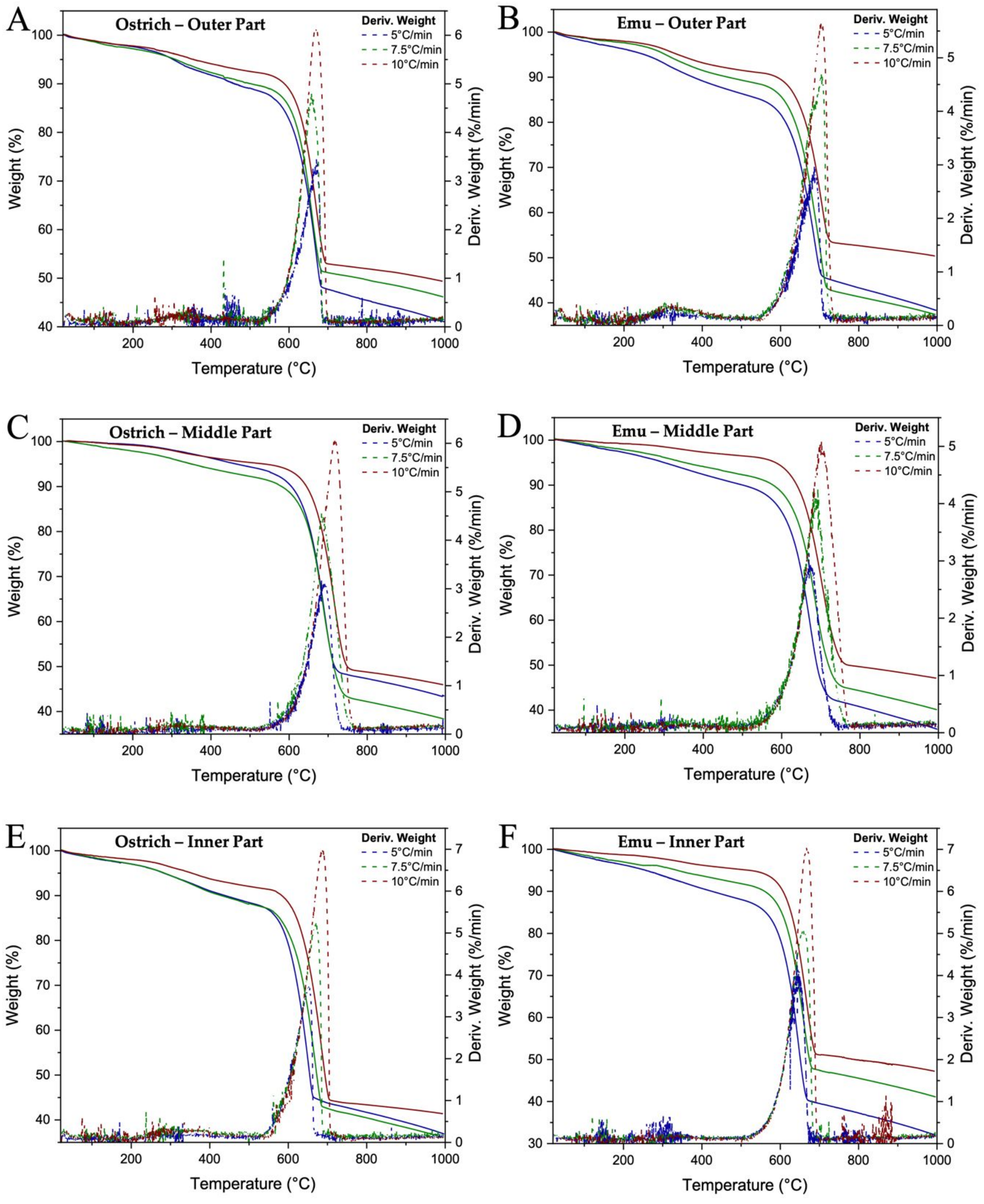

Figure 5. TG-DTG graphs of the outer, middle, and inner parts of eggshells of ratite birds.

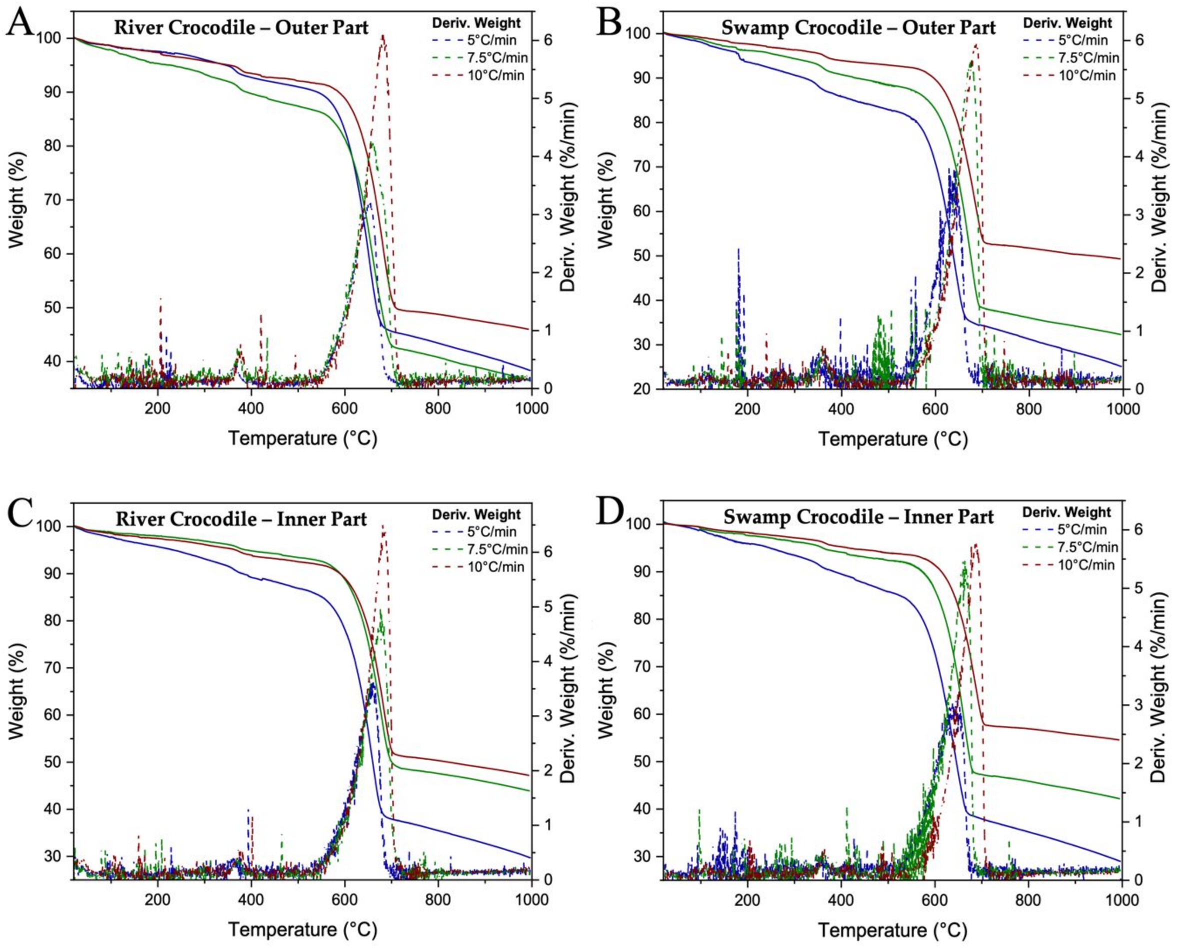

Figure 6. TG-DTG graphs of the outer, middle, and inner parts of eggshells of crocodiles.

Table 7. Maximum temperatures (TM) of DTG curves of ratite and crocodile eggshells at different heating rates. O: Outer; M: Middle; I: Inner*

	**Ratite Birds**						**Crododiles**			
	**Ostrich**			**Emu**			**River**		**Swamp**	
**T (ºC)**	**O**	**M**	**I**	**O**	**M**	**I**	**O**	**I**	**O**	**I**
5	670.16	691.00	653.41	687.69	674.45	645.87	653.46	661.05	638.28	645.87
7.5	658.78	685.53	670.47	704.12	689.63	657.26	659.27	676.25	676.24	663.06
10	668.50	717.52	687.58	702.80	702.81	666.64	680.03	682.04	685.84	685.84

*The data are given in ºC
